# Influenza vaccines and influenza antiviral drugs in Africa: are they available and do guidelines for their use exist?

**DOI:** 10.1186/1471-2458-14-41

**Published:** 2014-01-16

**Authors:** Jazmin Duque, Meredith L McMorrow, Adam L Cohen

**Affiliations:** 1 International Epidemiology and Research Team, Epidemiology Branch, Influenza Division, U.S. Centers for Disease Control and Prevention, 1600 Clifton Rd NE MS-A32, Atlanta, GA 30333, USA; 2 Battelle Atlanta, 2987 Clairmont NE Suite 450, Atlanta, GA 30329, USA; 3 U.S. Public Health Service, Rockville, MD, USA; 4 Influenza Program, U.S. Centers for Disease Control and Prevention-South Africa, PO Box 9536, Pretoria 0001, Republic of South Africa

**Keywords:** Influenza, Vaccine, Antiviral, Drugs, Policy, Recommendations, Africa

## Abstract

**Background:**

Influenza viruses cause significant morbidity and mortality in Africa, particularly among high-risk groups, but influenza vaccines and antiviral drugs may not be commonly available and used. The main aim of this study was to determine the availability and use of influenza vaccines and antiviral drugs as well as to describe existing related guidelines and policies in Africa.

**Methods:**

A self-administered survey was distributed among key influenza experts in 40 African countries.

**Results:**

Of the 40 countries surveyed, 31 (78%) responded; 14/31 (45%) reported availability of seasonal influenza vaccine, and 19/31 (65%) reported availability of antiviral drugs for the treatment of influenza. Vaccine coverage data were only available for 4/14 (29%) countries that reported availability of seasonal influenza vaccine and ranged from <0.5% to 2% of the population.

**Conclusions:**

Influenza vaccines and antiviral drugs are available in many countries in Africa but coverage estimates are low and remain largely unknown. Describing the local burden of disease and identifying funding are essential to encourage countries to use influenza vaccine more widely.

## Background

Influenza is an important contributor to morbidity and mortality in Africa [1]. Among 15 countries of the African Network for Influenza Surveillance and Epidemiology (ANISE), 10% and 22% of inpatient and outpatient respiratory cases, respectively, tested positive for influenza between 2006–2010 [2]. For many years, influenza epidemiology has been described in countries with temperate climates like South Africa and Morocco [3,4] but there are now data that comprehensively describe influenza viruses in tropical countries like Kenya and Zambia [5,6]. In Africa, influenza causes severe illness and deaths in both temperate and tropical settings [7,8].

Populations in low and middle income countries like many of those in Africa are more vulnerable to influenza-related complications because of the high prevalence of underlying medical conditions and limited access to health care. For example, outbreaks of influenza A(H3N2), which circulates widely across the globe, have caused unusually high case fatality ratios in Madagascar and the Democratic Republic of Congo [9,10]. In addition, the elderly in South Africa are four times more likely to die from an influenza infection than their counterparts in the United States [11]. The high prevalence of comorbidities including human immunodeficiency virus and tuberculosis contribute to increased influenza-associated mortality in Africa [7].

Vaccination is the most effective way to prevent influenza illness and antiviral drugs help treat viral infection [12]. Vaccines and antiviral drugs are important particularly among high risk groups such as young children, pregnant women, the elderly and persons with underlying medical conditions. The World Health Organization (WHO), however, reports that none of the countries in the African Region have capacity to produce seasonal influenza vaccines [13] and that only 2 of the 54 countries on the continent have access to them [14]. A separate report from the International Federation of Pharmaceutical Manufacturers states that countries in Africa, the Eastern Mediterranean and South-East Asia combined receive only 1% to 4% of the global seasonal influenza vaccine supply each year [15]. With growing data on the burden of influenza across the African continent, many countries are considering introducing or expanding strategies to prevent and manage influenza infection. We conducted this study to gather more data on the availability of influenza vaccines and antiviral drugs in Africa as well as related national policies and guidelines.

## Methods

The U.S Centers for Disease Control and Prevention (CDC) has long-standing relationships with Ministries of Health, WHO, Institut Pasteur and the U.S. Department of Defense. We approached these institutions to identify key in-country influenza experts in Africa who could answer the survey. In addition, La Réunion, a French territory in the Indian Ocean east of Madagascar, was asked to participate. Since La Réunion is not a country, their data were excluded from the country analyses and are presented separately. This study was considered to be an evaluation of existing public health programs and not considered to be public health research.

The survey included 22 questions regarding influenza vaccines, antiviral drugs and related policies and guidelines and was administered in both English and French. We created an electronic version of this questionnaire using SurveyMonkey© (SurveyMonkey, Palo Alto, CA) and collected responses, either electronically or in hard copy form, from January 2012 through January 2013. Surveys were distributed to representatives from 24 countries attending the Third Annual ANISE Meeting in Nairobi, Kenya [16], via email and in person. Data were entered and analyzed using Microsoft Access© (Microsoft Corporation, Redmond, WA) and OpenEpi [17].

In some cases, more than one person responded per country. In instances in which survey responses did not match, the response from the representative from the Ministry of Health or equivalent was considered valid. If there was no clear valid response, we tried to determine a valid response via personal communication with an in-country influenza expert.

## Results

Of the 54 countries on the African continent, we contacted 40 (74%) for this study, of which 31 (78%) completed the survey. There were 2 or more respondents for 12/31 (39%) of the countries represented. A representative from La Réunion also completed the survey.

**Figure 1:**
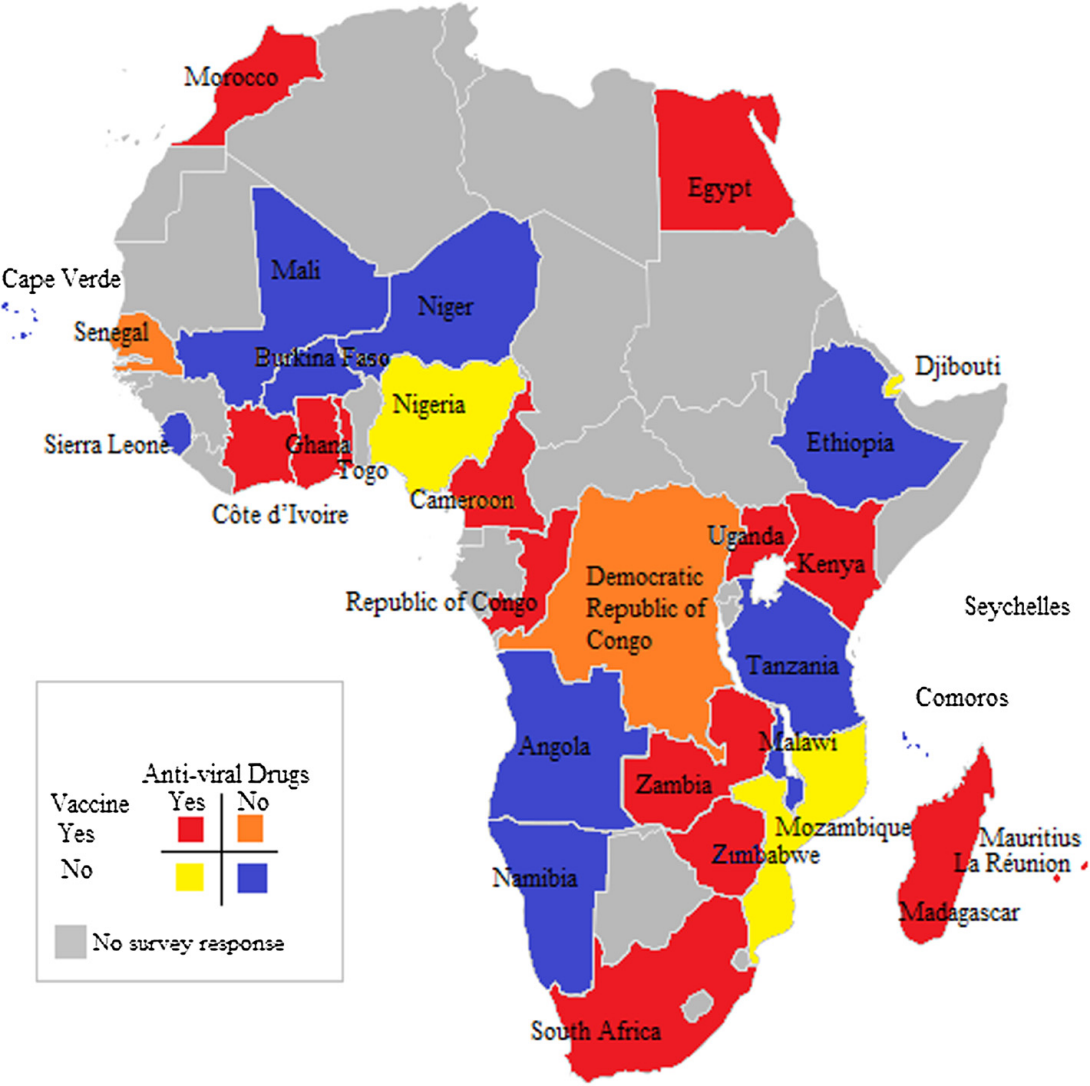
Availability of influenza vaccine and antiviral drugs in Africa.

Of the 31 countries surveyed, 14 (45%) reported availability of seasonal influenza vaccine (Figure 1); six in the private sector only (Democratic Republic of Congo, Senegal, Togo, Uganda, Zambia, and Zimbabwe) and eight in both private and public sectors (Cameroon, Côte d’Ivoire, Egypt, Kenya, Madagascar, Mauritius, Morocco, and South Africa) (Table 1). Moreover, although we were not able to find anyone to respond to this survey in Algeria, WHO reports influenza vaccine is available there [14]. La Réunion also reported availability of vaccine in both the private and public sectors.

**Table 1:** Availability of influenza vaccine and antiviral drugs for the treatment of influenza in Africa*

**Country**	**Region**	**World Bank income level**	**Vaccine available**	**Sector (Public vs private)**	**Type of vaccine**	**Antiviral drugs available**	**Sector (Public vs private)**
Angola	South	Upper middle	No	--	--	No	--
Burkina Faso	West	Low	No	--	--	No	--
Cameroon	Central	Lower-middle	Yes	Both	Southern	Yes	Public
Cape Verde	West	Lower-middle	No	--	--	No	--
Comoros	Indian Ocean	Low	No	--	--	No	--
Côte d’Ivoire	West	Lower-middle	Yes	Both	Northern	Yes	Public
Democratic Republic of Congo	Central	Low	Yes	Private	Northern	No	--
Djibouti	East	Lower-middle	No	--	--	Yes	Public
Egypt	North	Lower-middle	Yes	Both	Unknown	Yes	Both
Ethiopia	East	Low	No	--	--	No	--
Ghana	West	Lower-middle	No	--	--	Yes	Public
Kenya	East	Low	Yes	Both	Southern	Yes	Private
Madagascar	Indian Ocean	Low	Yes	Both	Southern	Yes	Both
Malawi	South	Low	No	--	--	No	--
Mali	Central	Low	No	--	--	No	--
Mauritius	Indian Ocean	Upper-middle	Yes	Both	Southern	Yes	Both
Morocco	North	Lower-middle	Yes	Both	Northern	Yes	Both
Mozambique	South	Low	No	--	--	Yes	Private
Namibia	South	Upper-middle	No	--	--	No	--
Niger	Central	Low	No	--	--	No	--
Nigeria	West	Lower-middle	No	--	--	Yes	Public
Republic of Congo	Central	Lower-middle	No	--	--	Yes	Both
Réunion (France)	Indian Ocean	NA	Yes	Both	Southern	Yes	Both
Senegal	West	Lower-middle	Yes	Private	Northern	No	--
Seychelles	Indian Ocean	Upper-middle	No	--	--	Yes	Public
Sierra Leone	West	Low	No	--	--	No	--
South Africa	South	Upper-middle	Yes	Both	Southern	Yes	Both
Tanzania	East	Low	No	--	--	Yes	Both
Togo	West	Low	Yes	Private	Unknown	Yes	Both
Uganda	East	Low	Yes	Private	Southern	Yes	Private
Zambia	South	Lower-middle	Yes	Private	Unknown	Yes	Private
Zimbabwe	South	Low	Yes	Private	Unknown	Yes	Both

*This table includes La Réunion (a French territory in the Indian Ocean) in addition to 31 African countries.

Regarding the composition of vaccine used, six countries reported using the Southern Hemisphere vaccine, four the Northern Hemisphere, and four were unknown. La Réunion reported using Southern Hemisphere vaccine as well (Table 1). Vaccine coverage data were only available for 4/14 (29%) countries that reported availability of seasonal influenza vaccine and ranged from <0.5% to 2% of the population. Countries that had influenza vaccine available were from all regions of Africa and represented all World Bank income classifications, except high income as there are no high income countries in Africa (Table 1) [18]. Of note, vaccine availability was not associated with World Bank income level (chi-square = 0.19; p-value = 0.57).

Among the 14 countries that reported having any influenza vaccine, four (Côte d’Ivoire, Egypt, Mauritius, and Morocco) reported having a national public policy for its use; all of them target healthcare workers, young children, the elderly, and persons with underlying medical conditions. Three target pregnant women and one pilgrims going to Hajj. South Africa has standing guidelines for the prevention and treatment of influenza [19]but a national public policy regarding vaccination is yet to be developed.

Nine respondent countries felt national decision-makers would be willing to introduce vaccine, of which six (67%) (Angola, Mozambique, Nigeria, Seychelles, Togo, and Zimbabwe) said the country could do so within the next 4 years. These same respondents ranked demonstration of public health burden within the country and availability of international funding as most important in influencing decision-makers to introduce and promote influenza vaccine; evidence that illnesses and deaths can be prevented by influenza vaccination and evidence that the national health system will have a cost-benefit with the introduction of vaccine were still considered important but did not rank as high.

Of the 31 respondent countries, 23 (74%) reported receiving and/or purchasing pandemic influenza vaccine in or after 2009, and 19 (83%) of these reported using these vaccines. Four countries (Kenya, Mali, Senegal and South Africa) and La Réunion reported conducting influenza vaccine research in the years 2012 and/or 2013.

Lastly, 19 (65%) of the 31 respondent countries reported availability of antiviral drugs for the treatment of influenza; nine in both the public and private sector, six in the public sector only and four in the private sector only (Table 1). La Réunion also reported availability of antiviral drugs in both the private and public sectors. Cameroon, Egypt, Ghana and South Africa reported having national guidelines for the use of these antiviral drugs.

## Discussion and conclusions

Influenza vaccines and antivirals are available in Africa although population coverage is estimated to be very low. Vaccines and antivirals are available in the private sector alone or both private and public sectors. The Southern Hemisphere formulation is most widely used in Africa despite lower production worldwide [13]. Over the last few years, the Northern and Southern hemisphere influenza vaccine formulations have been identical albeit the time in which these vaccines are distributed is different. Several countries that do not currently have influenza vaccine available are likely to consider doing so in the next few years and would use national burden estimates to inform policy. However, funding is a concern. As has been seen globally with other underutilized vaccines [20], many of the countries in Africa without influenza vaccine are low income. The Global Alliance for Vaccines and Immunisation (GAVI) is a public-private partnership that aids low income countries in procuring vaccines. Currently, there are 56 countries eligible to receive GAVI support and 35 of these countries are in Africa [21].

Before the 2009 influenza A(H1N1) pandemic, most countries in Africa had inadequate data on the burden of influenza disease and high risk groups to inform influenza public health policies [22]. Since then, influenza surveillance systems have been strengthened and many countries are studying influenza incidence and seasonality, particularly among sub-groups [1,2]. Networks like ANISE [2] and AfriFlu [23] have increased the scientific dialogue among influenza experts in Africa.

Globally, one of the main concerns is whether seasonal influenza vaccine supply is adequate to meet the needs of all countries for future pandemics [13,24,25]. A survey among 10 countries in Southeast Asia, where influenza pandemics are likely to emerge, found seasonal influenza vaccine sales in the private sector average <1000 doses per 100,000 population and guidelines for vaccine use in only half of these countries [20]. In contrast, 5/15 (33%) of the African countries with influenza vaccine reported having national guidelines for influenza vaccines. Investment in influenza research to promote evidence-based influenza public health policies in Africa is essential to encouraging policy development and implementation.

The main limitation of this study is that these findings, albeit reported by credible sources, may not accurately reflect the true vaccine and antiviral use in Africa, especially since we were unable contact representatives in 14 countries and did not get a response from 9 countries, many of which are lower income. It is very difficult in many of these countries, for example, to ascertain vaccine coverage rates and to understand vaccine supply. While vaccines may be available, distribution and use by high risk groups remains unknown. Given that such a small proportion (1%-4%) of the global seasonal vaccine supply is distributed in Africa, the Middle East, and Southeast Asia [15], and since most of the world’s population lives in these areas, vaccination rates must be low. As additional data emerge on the burden of influenza disease in Africa, the identification of groups at risk of serious complications must be met with increasing availability of vaccines, policies targeting identified risk groups, and funding to support larger scale procurement and distribution efforts in resource-poor countries.

## Abbreviations

ANISE: African Network for Influenza Surveillance and Epidemiology; CDC: U.S. Centers for Disease Control and Prevention; WHO: World Health Organization.

## Competing interests

The authors declare they have no competing interests.

## Authors’ contributions

JD contributed to the study conception, design, data acquisition, data analysis and writing of this manuscript. MM contributed to the acquisition of the data and writing of this manuscript. AC contributed to the study conception, design, data acquisition, data analysis and writing of this manuscript. All authors read and approved the final manuscript.

## Pre-publication history

The pre-publication history for this paper can be accessed here:

http://www.biomedcentral.com/1471-2458/14/41/prepub
